# Dynorphin A induces membrane permeabilization by formation of proteolipidic pores. Insights from electrophysiology and computational simulations

**DOI:** 10.1016/j.csbj.2021.12.021

**Published:** 2021-12-16

**Authors:** D. Aurora Perini, Marcel Aguilella-Arzo, Antonio Alcaraz, Alex Perálvarez-Marín, María Queralt-Martín

**Affiliations:** a Laboratory of Molecular Biophysics. Department of Physics. Universitat Jaume I, 12071 Castellón, Spain; b Biophysics Unit, Department of Biochemistry and Molecular Biology, School of Medicine, Universitat Autònoma de Barcelona, 08193 Cerdanyola del Vallès, Spain; c Institute of Neuroscience, Universitat Autònoma de Barcelona, 08193 Cerdanyola del Vallès, Spain

**Keywords:** Dynorphin, Membrane permeabilization, Ion channel, Noise and fluctuations, Protein-lipid interactions, Proteolipidic pores, Computational simulations

## Abstract

Dynorphins are endogenous neuropeptides that function as ligands for the κ-opioid receptor. In addition to opioid activity, dynorphins can induce several pathological effects such as neurological dysfunctions and cell death. Previous studies have suggested that Dynorphin A (DynA) mediates some pathogenic actions through formation of transient pores in lipid domains of the plasma membrane. Here, we use planar bilayer electrophysiology to show that DynA induces pore formation in negatively charged membranes. We find a large variability in pore conformations showing equilibrium conductance fluctuations, what disregards electroporation as the dominant mechanism of pore formation. Ion selectivity measurements showing cationic selectivity indicate that positive protein charges of DynA are stabilized by phosphatidyl serine negative charges in the formation of combined structures. We complement our study with computational simulations that assess the stability of diverse peptide arrangements in the hydrophobic core of the bilayer. We show that DynA is capable of assembling in charged membranes to form water-filled pores that conduct ions.

## Graphical abstract

**Figure:**
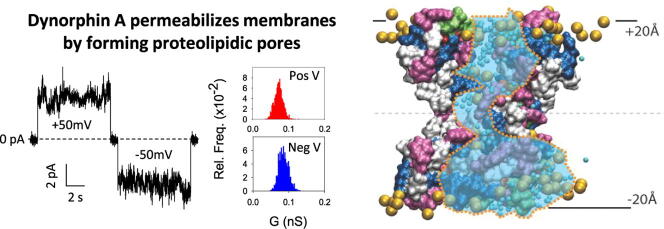

## 1 Introduction

Dynorphins are prohormone opioid endogenous peptides derived from prodynorphin (PDYN) [1], whose expression is altered in brain of drug/alcohol abusers and neurological disorder patients [2], [3], [4], [5]. PDYN is cleaved at positively charged residue motifs by proprotein convertase 2 and other enzymes yielding shorter intermediates, such as, big dynorphin (BigDyn, 32 residues). BigDyn is further processed into Dynorphin A (DynA, 17 residues) and Dynorphin B (DynB, 13 residues) [1], two of the most basic peptides found in the human body, which are the canonical substrate for the kappa-opioid receptor [6], but also pathological ligands for NMDA-R [5], [7] and ASIC1a [8], [9], [10].

Beyond the opioid effects, DynA has been implicated in several other signaling off-pathways with a plethora of pathological effects, including paralysis and death of neural cells [11], [12], [13]. DynA as a highly positive peptide in humans [14], [15], partitions and changes its secondary structure with negatively charged molecules, like detergents [16] and lipids [17], [18], [19]. Studies on DynA shorter analogues [20] and clinical variants [21], [22] have revealed peptide secondary structure and bilayer partition propensities. In fact, pathophysiological mechanisms for DynA have been described in relation to Ca^2+^-leakage due to its potential cell penetrating peptide (CPP)-like behavior [18], [23]. CPPs are positively short peptides that are capable of translocating themselves and also deliver a wide variety of cargos into cells [24]. Despite having outstanding potential applications in biomedicine and pharmacology, no CPPs or CPP/cargo complexes have been approved yet for clinical use [25], probably because the necessary understanding of how CPPs challenge membrane impermeability is yet to emerge. Here, we focus on how DynA peptides interact with each other and with lipid membranes, having in mind that several models have been proposed including spontaneous pore-formation (barrel-stave, toroidal or arch pores [26], [27]) and also electroporation as direct consequence of the presence of a transmembrane voltage [28]. Alternatively, “detergent-like mechanisms” are invoked when proteins just disrupt the lipid packing and disintegrate the bilayer without showing reproducible conductive patterns [27], [29].

Experimentally, the membrane disruption and translocation potential of dynorphins has been previously studied by using different techniques such as by circular dichroism, nuclear magnetic resonance (NMR) spectroscopy [21] and confocal fluorescence microscopy/immunolabeling [23] among others [16], [17], [18], [19], [21], [22], [23]. The potential pore formation propensities were studied using electrophysiology of DRG neurons [30]. Big dynorphin was the most active translocating and pore-forming peptide yielding giant pores of ca. 3 nm [30], albeit DynA was not studied in such detail and its mechanism of action is yet to be understood. In this work we investigate the membrane permeabilization induced by DynA in planar bilayers formed by negatively charged phospholipids. Electrophysiology in model membranes is particularly useful to search for minimal conductive units (single channel conductance), investigate the presence of large conductive pores as opposed to the simultaneous action of clusters of small conductive pores [31] and also discriminate between equilibrium and non-equilibrium pore formation mechanisms by performing noise analysis on the recorded currents [32].

To counterpart electrophysiology, we benefit here from biophysical modelling by performing Molecular Dynamics simulations. In particular, we model potential molecular systems *in silico* combining coarse-grain and all-atom simulations to shed light into the molecular details of the DynA pore formation to understand the off-pathway pathophysiology of dynorphin neuropeptides. Computational biophysics is becoming an important tool for the study of pore forming potential of peptides in bilayers because it helps visualizing and understanding molecular details [33]. Current computational developments in lipid parameters and force fields [34], [35], and combination of coarse grain and all-atom simulations to overcome computational costs [36], are being implemented to expand our knowledge on peptide-lipid interactions, pore nature and organization, and stability of the complexes, to gain insight into peptide-induced membrane perturbation effects [37].

## 2 Material and methods

### 2.1 Planar lipid membrane formation

Planar membranes were formed by using a solvent-free modified Montal-Mueller technique [38], [39]. In brief, the lipid was prepared by dissolving diphytanoyl-phosphatidylserine (DPhPS) (Avanti polar lipids, Inc., Alabaster, AL) in pentane at 5 mg/ml after chloroform evaporation. Aliquots of 10–20 µl of lipid in pentane were added onto salt solution subphases buffered with HEPES 5 mM at pH 7.4 in two 1.6 ml compartments (so-called *cis* and *trans*) of a Teflon chamber. The two compartments were separated by a 15 µm-thick Teflon film with a 70–100 µm diameter orifice. The orifices were pre-treated with a 1% solution of hexadecane in pentane. After pentane evaporation, the level of solutions in each compartment was raised above the hole so the planar bilayer could form by apposition of the two monolayers.

### 2.2 DynA-induced current measurements

DynA peptides were purchased from Pepmic Co. (Suzhou, China) in powder and dissolved in MilliQ® water. DynA-induced currents were achieved by adding 1 µl of a 500 µg/ml solution of DynA peptides close to the partition from the CIS side of the chamber, which corresponds to a DynA peptide final concentration of 0.31 µg/ml or 195 nM. This is a concentration unlikely to drive spontaneous hydrolysis, especially when compared to other *in vitro* studies using µm-mM range DynA concentrations [16], [17], [18], [19]. After protein addition, membrane was reformed several times until DynA-induced currents were observed. An electric potential was applied using Ag/AgCl electrodes in 2 M KCl, 1.5% agarose bridges assembled within standard 250 µl pipette tips. The potential was defined as positive when it was higher on the side of peptide addition (*cis* side), whereas the *trans* side was set to ground. An Axopatch 200B amplifier (Molecular Devices, Sunnyvale, CA) in the voltage-clamp mode was used to measure the current and the applied potential. Current was filtered with a 10 kHz 8-pole in-line Bessel filter and digitized with a Digidata 1440A (Molecular Devices, Sunnyvale, CA) at 50 kHz sampling frequency. The membrane chamber and the head stage were isolated from external noise sources with a double metal screen (Amuneal Manufacturing Corp., Philadelphia, PA). The conductance was obtained from current measurements under an applied potential of 50 mV in symmetrical salt solutions of 150 mM KCl buffered with 5 mM HEPES at pH 7.4. The conductance values were evaluated using the Gaussian fit tool of Clampfit 10.7 (Molecular Devices, Sunnyvale, CA). Absolute conductance values were obtained from any type of events, either noisy and flickering or more stable currents, as long as they can be represented by a Gaussian fitting. Conductance increment values were calculated analyzing step-wise events of increase or decrease of current, regardless of their stability or duration.

### 2.3 Ion selectivity measurements

Cation vs. anion preference of DynA-induced currents was assessed by measuring the reversal potential (RP), the applied voltage needed to cancel the current measured when a salt concentration gradient is imposed in the system. Planar membranes were formed under 5-fold (100 mM/500 mM KCl) and 10-fold (100 mM/1 M KCl) concentration gradients and the net ionic current obtained was manually set to zero by adjusting the applied potential. This potential was then corrected by the liquid junction potential of the electrode salt bridges [40] to obtain the RP. The measured RP was converted into channel permeability (P_+_/P_-_) by means of the Goldman–Hodgkin–Katz (GHK) [41] equation.

### 2.4 Current fluctuation analysis

The power spectral density (PSD) of current fluctuations was obtained directly from the measured current traces with the pClamp 10.7 software (Molecular Devices, LLC.). The power spectrum generates a frequency domain representation of the time domain data, revealing the power levels of different frequency components in the signal. PSD was measured by calculating the Fast Fourier Transform from the digitized signal after application of a 1 kHz 8-pole Bessel lowpass digital filter. The PSD spectral resolution used was 0.76 Hz and, for each signal, the available spectral segments were averaged. PSD voltage-dependence was assessed by averaging in the 1–10 Hz band the obtained PSDs at each applied potential.

### 2.5 Computational methods – system setup

The initial DynA peptides were modelled using the i-Tasser web service (https://zhanglab.dcmb.med.umich.edu/I-TASSER/) showing good agreement with previous published structures [16]. The resulting peptide model was minimized and equilibrated in aqueous solution and subsequently the resulting structures were used to prepare tentative configurations of 6 and 12 peptides using VMD [42]. The structure with 6 peptides (6 DynA) was arranged parallel to the membrane plane approximately in the center of the lipid bilayer. The initial structure of 12 DynA peptides (12 DynA) was arranged in a double barrel configuration with an inner diameter of approximately 13 Ångstroms, and peptides were positioned in the core of the bilayer perpendicular to the membrane plane. In all cases, the DynA peptide structures were inserted into a DPPS membrane using Charmm-Gui [43], adding water and ions to a concentration of 0.1 M and using the Martini22p coarse-grain (CG) force field with polarizable water [44]. The dimensions of each simulated system, number of water molecules, number of ions and total number of atoms can be found in Table 1.

**Table 1:** Details of the simulations of this study.

System	# Peptides	# Atoms	# Water molec.	# DPPS molec.	Dimensions (nm)	Time (µs)	T(K)
6 DynA (CGMD)	6	8638	2010	174	83x83x92	35	303.15
12 DynA (CGMD)	12	11071	2689	180	92x91x93	35	303.15
12 DynA (AAMD)	12	58348	10756	180	96x96x92	2	323.15

Minimization of 5000 Steepest Descent steps, and six equilibration steps (total time 4.75 ns) were performed keeping atoms, pressure and temperature constant (NPT) in which the restrictions over the protein and/or lipid were gradually released and the timestep gradually increased from 2 fs to 20 fs. After the minimization and equilibration processes, peptides had adopted different orientations with respect to the membrane plane (see Fig. 5A, leftmost panel (0 µs)). Then, a production step under the NPT ensemble was performed, extending the simulation to a minimum of 25 µs with a timestep of 20 fs. Simulations were run using GROMACS 2021 [45] on multiprocessor workstations with CUDA acceleration. In CG molecular dynamics (CGMD) simulations, Berendsen pressure coupling and reaction-field for electrostatics and a velocity rescale for the temperature coupling were used.

The 12 DynA final structure was converted to all atom (AA) using the “all-atom converter” tool in Charmm-Gui under the Charmm36m [46] force field. Simulations consisted of 5000 steepest descent minimization steps and six NPT equilibration steps in which the restrictions applied on the protein and membrane are released and the timestep gradually increased from 1 fs to 2 fs. Production step has been lengthened to a total of 2 µs for the 12 DynA system. In AA molecular dynamics (AAMD) simulations Parrinello-Rahman pressure coupling and Particle Mesh Ewald for electrostatics were used during the production step and Nose-Hoover for the temperature coupling. Temperature was increased in the AA simulation from 303.15 K to 323.15 K to challenge the robustness of the system.

## 3 Results and discussion

### 3.1 DynA induces formation of pores in negatively charged membranes

**Fig. 1:**
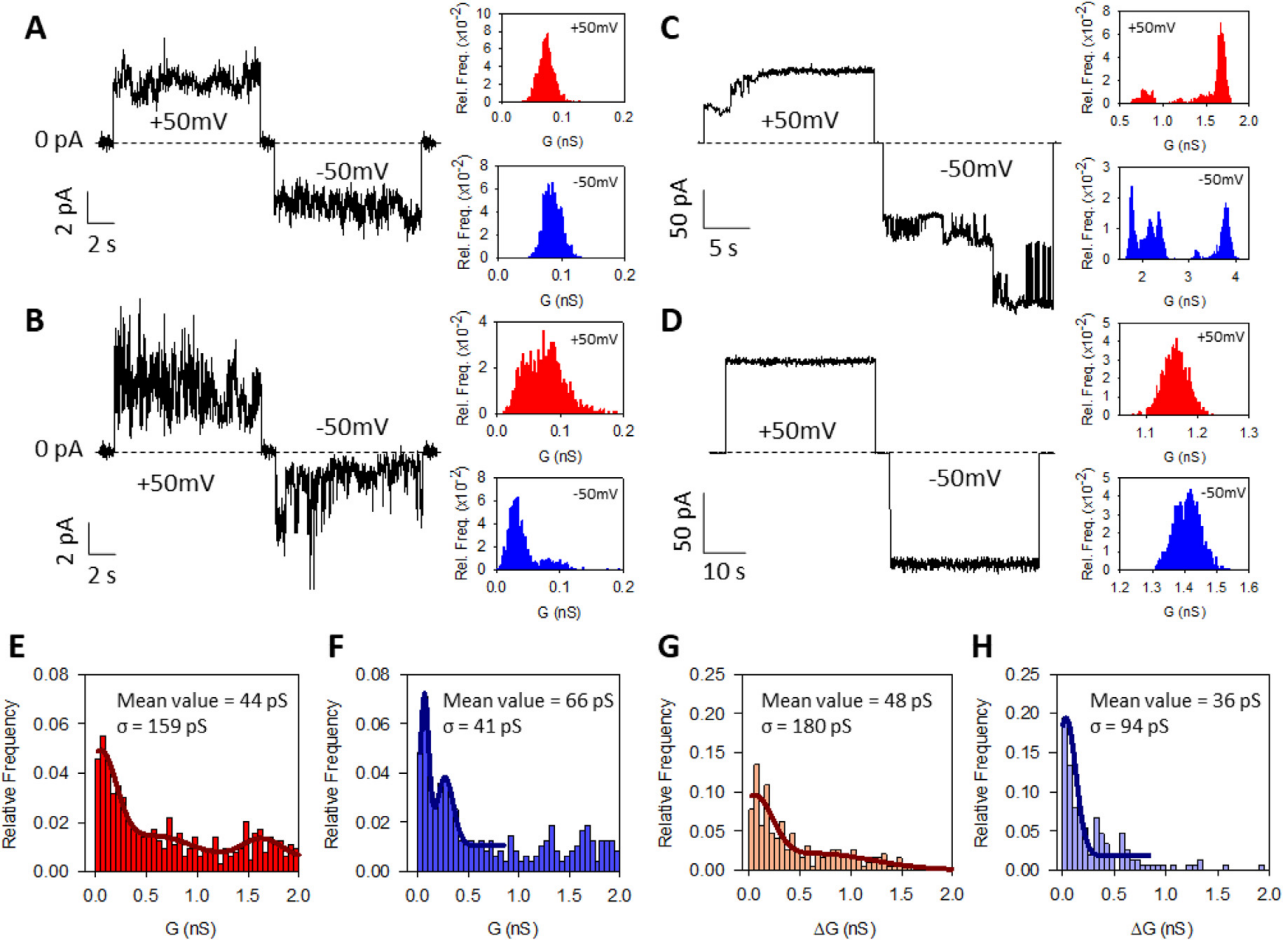
DynA induces formation of pores in negatively charged membranes. (A-B) Representative current versus time traces of DynA peptide showing small currents recorded at ±50 mV that can be represented by single peak histograms (A) or otherwise are noisy with fast flickering and occasional current bursts (B). (C-D) Representative current versus time traces of DynA peptide recorded at ±50 mV showing large currents with successive pore insertions (C) and stable currents with well-defined conducting levels and only minor current fluctuations (D). (E-H) Histograms of DynA-induced currents show the existence of a minimal conductive unit. Normalized histograms representing the absolute conductance levels (G (nS)) at positive (E) and negative (F) applied voltages. Conductance increments (ΔG (nS)) at positive (G) and negative (H) applied voltages. Histograms were built from 635 (E), 477 (F), 193 (G), and 149 (H) events. The absolute conductance fits show three peaks at positive voltage (44 ± 159 pS, 572 ± 359 pS and 1634 ± 194 pS) (E) and two peaks at negative applied voltage (66 ± 41 pS and 270 ± 81pS) (F). In each panel, the mean and SD is indicated for only the first peak. In all panels, salt solutions consisted of 150 mM KCl buffered with 5 mM HEPES at pH 7.4. Membranes were formed of DPhPS.

DynA, a protein soluble in water, was added to the solution surrounding a DPhPS bilayer (see Materials and Methods) in standard physiological conditions, 150 mM KCl, 5 mM HEPES pH 7.4. After some time, spontaneous protein insertions were observed, revealing ion channel activity with vast diversity of current levels and lifetimes, as shown in Fig. 1. Fig. 1A and Fig. 1B display representative traces corresponding to low conductive levels (∼50–100 pS). Despite having considerable noise, most traces can be represented by single peak histograms (right panels in Fig. 1A and Fig. 1B at V = -50 mV) or random transitions between multiple conducting levels (right panel in Fig. 1B at V = 50 mV).

DynA also induces much larger conductive levels (several nS), as shown in Fig. 1C and Fig. 1D. The current steps shown in Fig. 1C both at positive and negative applied voltages could come from successive insertions and/or retractions of independent small pores like those of Fig. 1A and Fig. 1B, but might also represent the evolution of much larger dynamical structures that change their pore size with the addition or subtraction of DynA peptides [32]. In contrast to Fig. 1C, traces also show frequently stable current sections with well-defined conducting levels and only minor current fluctuations that are compatible with quiet wide pores (Fig. 1D). The coexistence of conductive events showing typical ion channel features (vivid random transitions between levels as in Fig. 1C) and partial sections of traces showing much larger quiet “pores” that do not undergo spontaneous closures (Fig. 1D) fits in the context of the so-called “channel-pore dualism” [47], meaning that different mechanisms of membrane permeabilization could be operating simultaneously. However, our current recordings do not show signs of membrane disintegration: we do not find neither giant pores totally unresponsive to voltage nor the progressive current increase leading to membrane rupture characteristic of detergent-like mechanisms [29].

Histograms for each absolute conductance level G (∼500 events) recorded at positive and negative applied voltages are shown in Fig. 1E and Fig. 1F, respectively. In both polarities, the most probable conductance peak locates around G ∼ 50 pS and there are secondary peaks at higher conductances (several nS) suggesting a wide variety of pore conformations. Remarkably, such variability refers not only to static disorder (existence of different current levels) but also to dynamic disorder (diversity of lifetimes, data not shown) [48]. In order to discriminate between the collective action of clusters of small units (see Fig. 1A or Fig. 1B) and potential individual wide pores (Fig. 1C or Fig. 1D), we considered the conductance increments ΔG (Fig. 1G and Fig. 1H) associated to each individual current jump. Histograms of ΔG provide most probable values that are comparable to the absolute value of G obtained from absolute conductance level histograms (Fig. 1E,F). On the one side, this confirms the identification of the minimal conductive unit formed by DynA, with a conductance around G ∼ 50 pS. On the other side, the reiterative presence of large individual current jumps (G ≥ 1 nS) that do not lead to membrane rupture strongly points to the existence of individual pores with much larger radius than those minimal units of G ∼ 50 pS. As a first approximation, the pore conductance can be written as G ∼ κπr^2^/L where κ is the electrolyte conductivity (κ ∼ 1.8 S/m for KCl 150 mM KCl at pH 7.4) and L the pore length (the lipid bilayer is about 4 nm in length [49]). This allows for a rough estimation of the characteristic pore radius that would be r ∼ 0.25 nm for G ∼ 50 pS. The upper limit of our conductance measurements (G ∼ 2 nS) would correspond to r ∼ 1.5 nm.

**Fig. 2:**
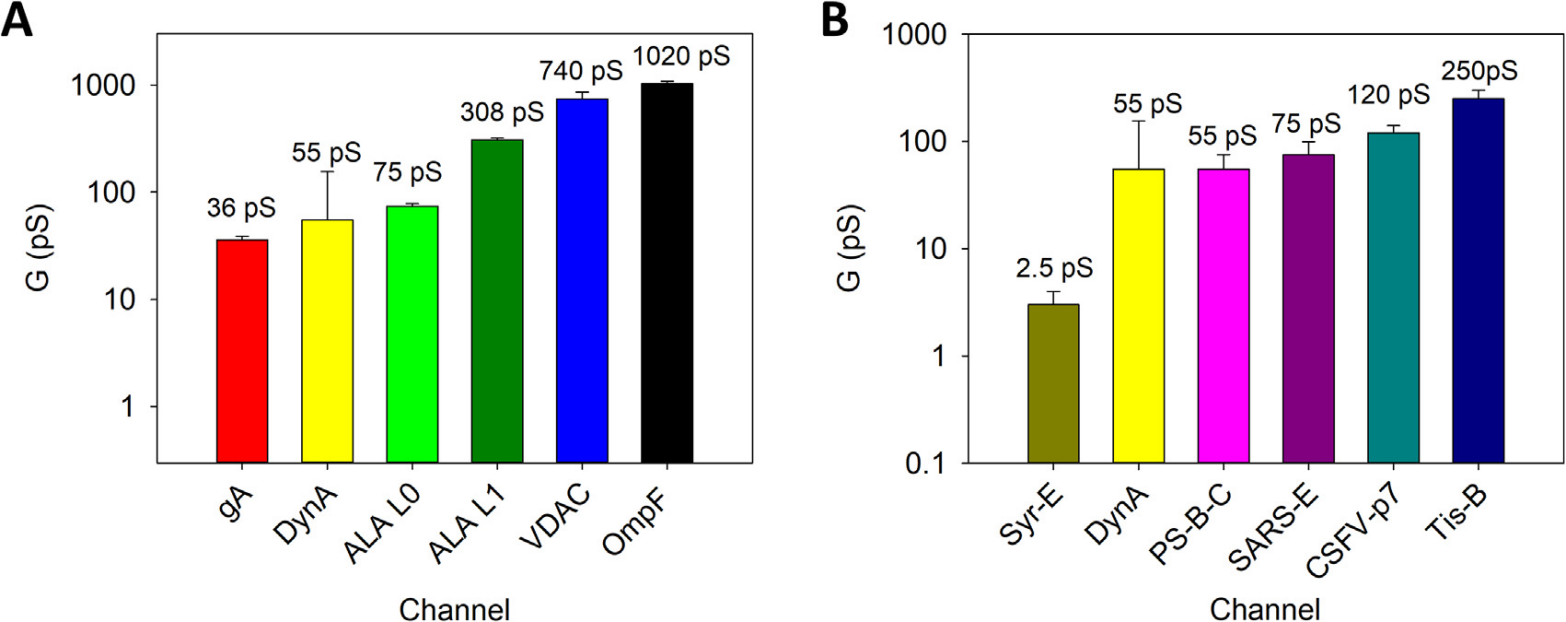
Comparison of single channel conductance of DynA with other channels of known dimensions in negatively charged membranes at 150 mM KCl and pH 7.4. (A) Comparison with protein ion channels. gA is Gramicidin A, ALA L0 and ALA L1 are the two conducting levels of Alamethicin, VDAC is the mitochondrial Voltage Dependent Anion Channel and OmpF is the Outer membrane protein F from E. Coli (B) Comparison with proteolipidic channels. Syr-E is Syryngomicyn-E (measurements in NaCl), Tis-B is a bacterial peptide, PS-B-C is a pulmonary surfactant hydrophobic protein, SARS-E is the SARS Coronavirus Envelope Protein, and CSFV-p7 is a Classical Swine Fever Virus protein. All values correspond to measurements conducted using DPhPS membranes, except for VDAC in which a Polar Lipid Extract with a ∼25% of negatively charged lipids was used.

Considering that DynA pores are probably not cylindrical and solution conductivity inside the pores is significantly different from that of the bulk due to nanoscale confinement [50], we can obtain an alternative pore sizing by comparing the minimal conductance obtained for DynA with those of the channels whose dimensions are already known for the same electrolyte conductivity. Fig. 2A shows measurements in DynA together with precedent studies conducted in negative planar lipid membranes on well-known channels such as Gramicidin A (gA) (r ∼ 0.4 nm)[51], Alamethicin with two conducting levels ALA-L0 (r ∼ 0.75 nm) and ALA-L1 (r ∼ 1.2 nm) [52], [53], the mitochondrial channel VDAC (r ∼ 1.25 nm) [54] and the trimeric bacterial porin OmpF (r ≤ 1 nm) [50], [55], [56]. As can be seen, DynA is somewhere between gA and ALA-L0, so that a minimal channel radius r ∼ 0.5 nm for DynA seems reasonable.

Also interesting is to compare the single channel conductance of DynA with other proteins that in combination with lipid molecules form proteolipidic channels, as shown in Fig. 2B. Antibiotic lipopeptide Syryngomicyn- E (Syr-E) [57] is considerably less conductive (by one order of magnitude) than DynA, which is, in contrast, comparable to the pores formed by hydrophobic proteins SP-B and SP-C of the pulmonary surfactant (PM-B-C) [31], the SARS Coronavirus Envelope Protein (SARS-E) [58], the p7 protein of the Classical Swine Fever Virus (CSFV-p7) [32], [59], [60] and the bacterial peptide Tis-B [61]. Although the actual architecture of the pores formed by proteins in Fig. 2 is still under debate, the fact is that lipid molecules are structurally and functionally involved in them, as seems to happen here with DynA that consistently forms channels is presence of charged lipids while only sporadic events are observed when added to membranes constituted by neutral ones (data not shown). This agrees with previous observations where DynA peptides induce membrane perturbation effects observed in partially negatively charged mixed phosphatidylcholine and phosphatidylglycerol compositions [18], [19].

### 3.2 Current-voltage relationships and noise analysis of DynA-induced pores

**Fig. 3:**
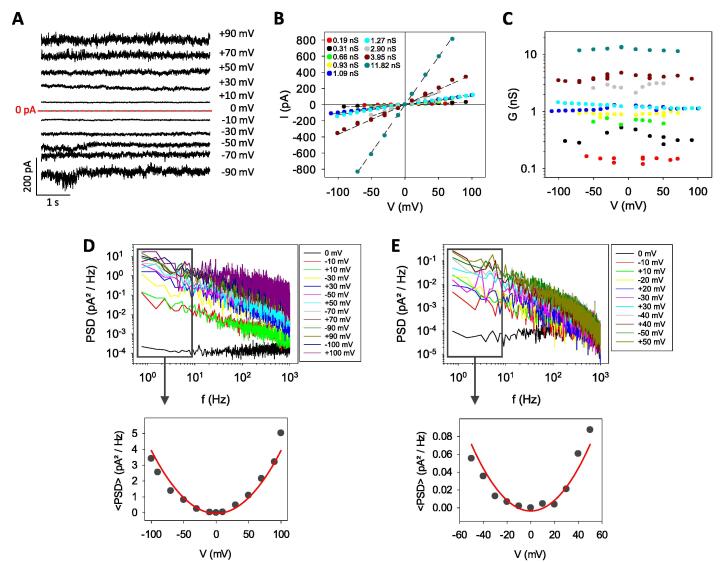
DynA-induced currents are ohmic for a wide range of measured conductances and characterized by 1/f PSDs displaying equilibrium conductance fluctuations. (A) Representative current trace (G = 3.95 nS) obtained from a stable DynA insertion and recorded at different applied voltages, as indicated. (B-C) Current-voltage (B) and conductance-voltage (C) curves obtained from stable DynA insertions with different absolute conductances. Data were obtained using 150 mM KCl buffered with 5 mM HEPES at pH 7.4 in DPhPS planar lipid membranes. (D-E) Upper panel: PSDs of two representative stable DynA-induced currents (G = 3.9 nS (D) and G = 0.9 nS (E)) at different applied voltages. PSDs are proportional to f^-α^, with α ∼ 0.8–1.0. Lower panel: Averaged PSDs obtained from the upper panel (1–10 Hz band), as a function of voltage. Solid lines represent a parabolic fitting. Data were obtained using 150 mM KCl buffered with 5 mM HEPES at pH 7.4 in DPhPS planar lipid membranes.

Next, the effect of the magnitude of the applied voltage on the channels formed by DynA was analyzed (note that Fig. 1 and Fig. 2 only contain measurements done under ± 50 mV). Fig. 3A shows representative stable current traces for different applied voltages. Although current fluctuations are intense, each current level still can be represented by a single peak in a histogram (Supplemental Fig. S1). Current-voltage curves in Fig. 3B show that the pore conductance is ohmic, similarly to proteolipidic systems such as SARS-E [56], [58] or CSFV-p7 [32], but in total contrast to others like Syr-E [57] or melittin [62], [63], [64] that show strongly voltage-dependent conductance. Fig. 3C. displays in detail the dependence of the channel conductance on the applied voltage, showing that the ohmic behavior of DynA-induced pores holds for more than two orders of magnitude of G values. Also, for a given channel, conductance does not depend neither on the magnitude of the applied voltage nor on its polarity. All together, these results indicate that the data presented in Fig. 1, Fig. 2 for measurements performed at ± 50 mV can be generalized to other voltage conditions.

Current traces contain other information than ionic conductance. Fig. 3A shows also that current oscillations increase with applied voltage making traces with higher currents to appear noisier. To examine this effect, the power spectral density (PSD) of DynA-induced currents at different applied voltages was calculated. The PSD quantifies the open channel current noise and provides the frequency hallmark [65] of the underlying physical mechanisms involved in pore formation. Fig. 3D-E shows the calculated PSDs of two independent DynA insertions of stable currents with an ohmic behavior over a wide voltage range (they correspond to the yellow and brown IV curves in Fig. 3B). The upper panel shows that all PSDs with V ≠ 0 scale as 1/f^α^, with α ∼ 0.8–1.0. 1/f^α^ spectrum is the most common outcome from noise analysis in the context of ion transport and it can be generated by many different mechanisms. Examples of processes that yield a 1/f^α^ spectrum are random transitions between open channel substates or between open and closed states [66], [67] and the existence of ion correlations [68]. Lower panels of Fig. 3D-E show that the PSD at low frequencies (1–10 Hz band) follow a parabolic dependence with the applied voltage. This is a distinctive feature of equilibrium conductance fluctuations [66], [69] and disregards electroporation as the dominant mechanism of pore formation by DynA [70].

### 3.3 Ionic selectivity of DynA-induced pores

**Fig. 4:**
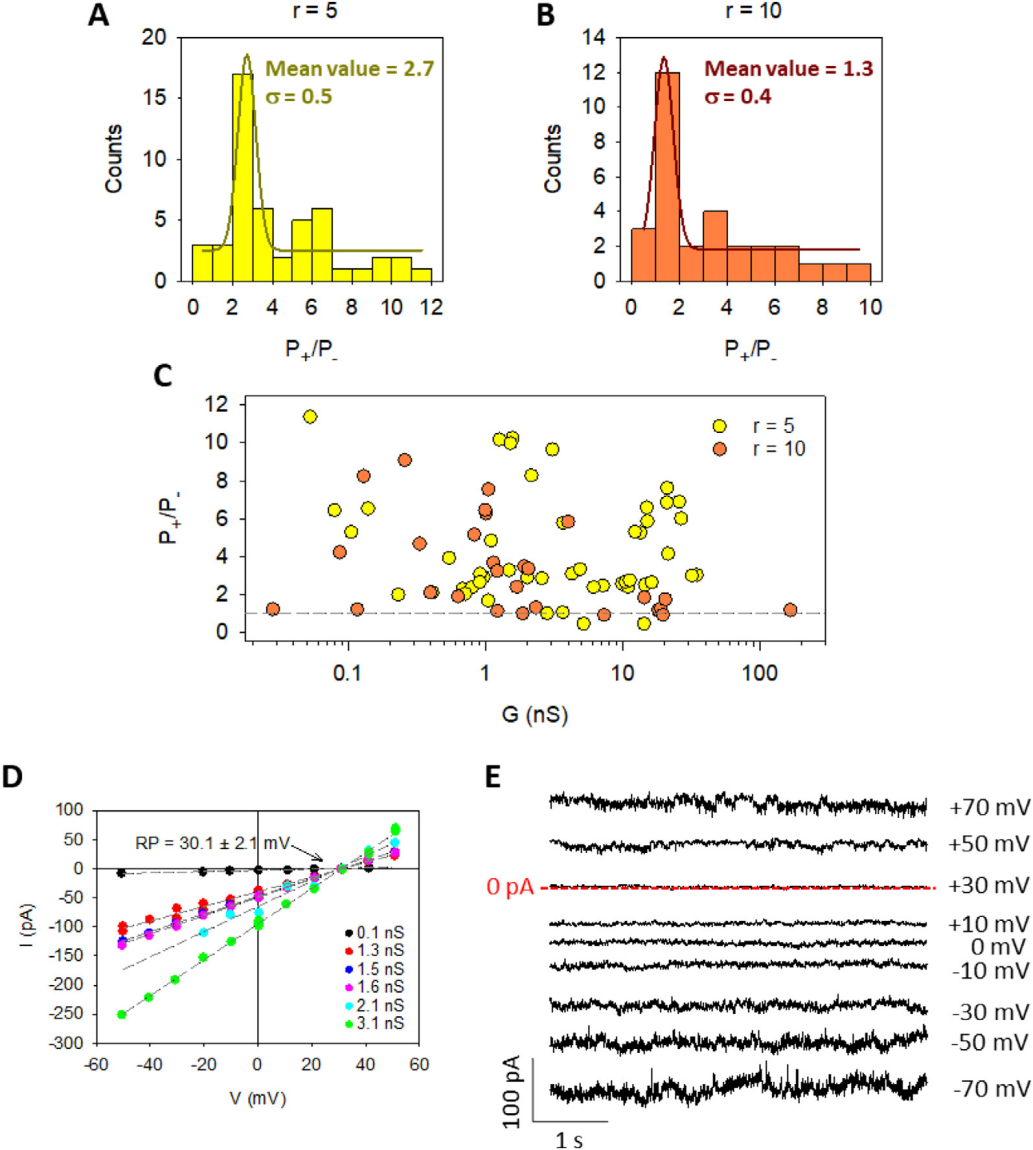
DynA displays a predominant selectivity to cations with some channels having different conductances but comparable selectivities. (A-B) Calculated permeability ratio P_+_/P_-_ from reversal potential experiments of DynA in 100 mM/500 mM KCl (gradient r = 5) (A) or in 100 mM/1 M KCl (gradient r = 10) (B) using DPhPS membranes. (C) Scatter plot of the permeability ratio as a function of the measured conductance. The dashed line represents P_+_/P_-_ = 1, which corresponds to a neutral channel. Data points above this line correspond to a cation selective pore, while those below the line reflect an anion selective channel. Data correspond to 49 (r = 10) and 30 (r = 5) independent DynA insertions. (D) Current-voltage curves obtained under a 5-fold salt gradient (100 mM/500 mM). (E) Representative traces of DynA current obtained under a 5-fold salt gradient (100 mM/500 mM).

The study of DynA pore formation was complemented with ion selectivity measurements by considering the voltage required to yield zero current under a transmembrane gradient, the so-called reversal potential (RP). The sign of the measured RP provides a quick estimation of the channel preference for anions or cations [71], but a more quantitative estimation is provided by the Goldman–Hodgkin–Katz (GHK) equation [41] that yields the permeability ratio P_+_/P_-_. Fig. 4 shows the permeability ratio for experiments carried out under 5-fold (100 mM/500 mM KCl, Fig. 4A) and 10-fold (100 mM/1 M KCl, Fig. 4B) concentration gradients.

In both cases the most probable values in the histogram are consistent with weak cationic selectivity, being P_+_/P_-_ = 2.7 ± 0.5 in Fig. 4A and P_+_/P_-_ = 1.3 ± 0.4 in Fig. 4B. Such mild discrimination is comparable to other proteolipidic pore systems like PS-B-C [31] or CSFV p7 [59] and suggests that positive protein charges of Dyn-A are stabilized by PS negative charges in the formation of a joint proteolipidic assembly [57]. Although the vast majority of pores seem to correspond to well-balanced arrangements between protein and lipid charges, we found some values of P_+_/P_-_ ∼ 5–10 corresponding to conformations with predominant lipid molecules and also very rarely (∼5% of the measurements), anion selective channels (P_+_/P_-_ < 1) in which lipid negative charges are outnumbered by protein basic residues.

Fig. 4C shows the corresponding conductance for each data point contributing to the RP histograms in Fig. 4A and Fig. 4B. RP measurements could be used to gain more insight about the pore size distribution. Generally, the wider the channel (and hence more conductive) the weaker the selectivity, because pore charges are further from permeating ions and electrostatic interactions are weakened [71], [72]. But, when dealing with identical pores, the measured RP does not depend on the number of inserted channels, so this can be used to detect the concerted action of a number of pores [71], [73]. The large dispersion found in Fig. 4C demonstrates that the connection between conductance and selectivity is not straightforward because large conductances may correspond to large pores, but also to clusters of small pores as discussed in Fig. 1. The latter possibility is depicted in Fig. 4D, that displays example current–voltage curves recorded under the same concentration gradient (100 mM/500 mM KCl). Despite each curve having a different slope (conductance), all of them share the same intercept (RP value), probably reflecting different multiplicities of the same (or very similar) channel configuration. Fig. 4E shows a representative current trace of these type of channels corresponding to G ∼ 3 nS. The RP ∼ 30 mV is clearly visible corresponding to zero current. At high applied voltages (see + 70 mV, for instance), small current fluctuations (ΔG ∼ 0.1 nS) reveal slight changes (spontaneous openings and closing) in the number of pore units.

### 3.4 Computational simulations of DynA-lipid interactions in bilayer membranes

**Fig. 5:**
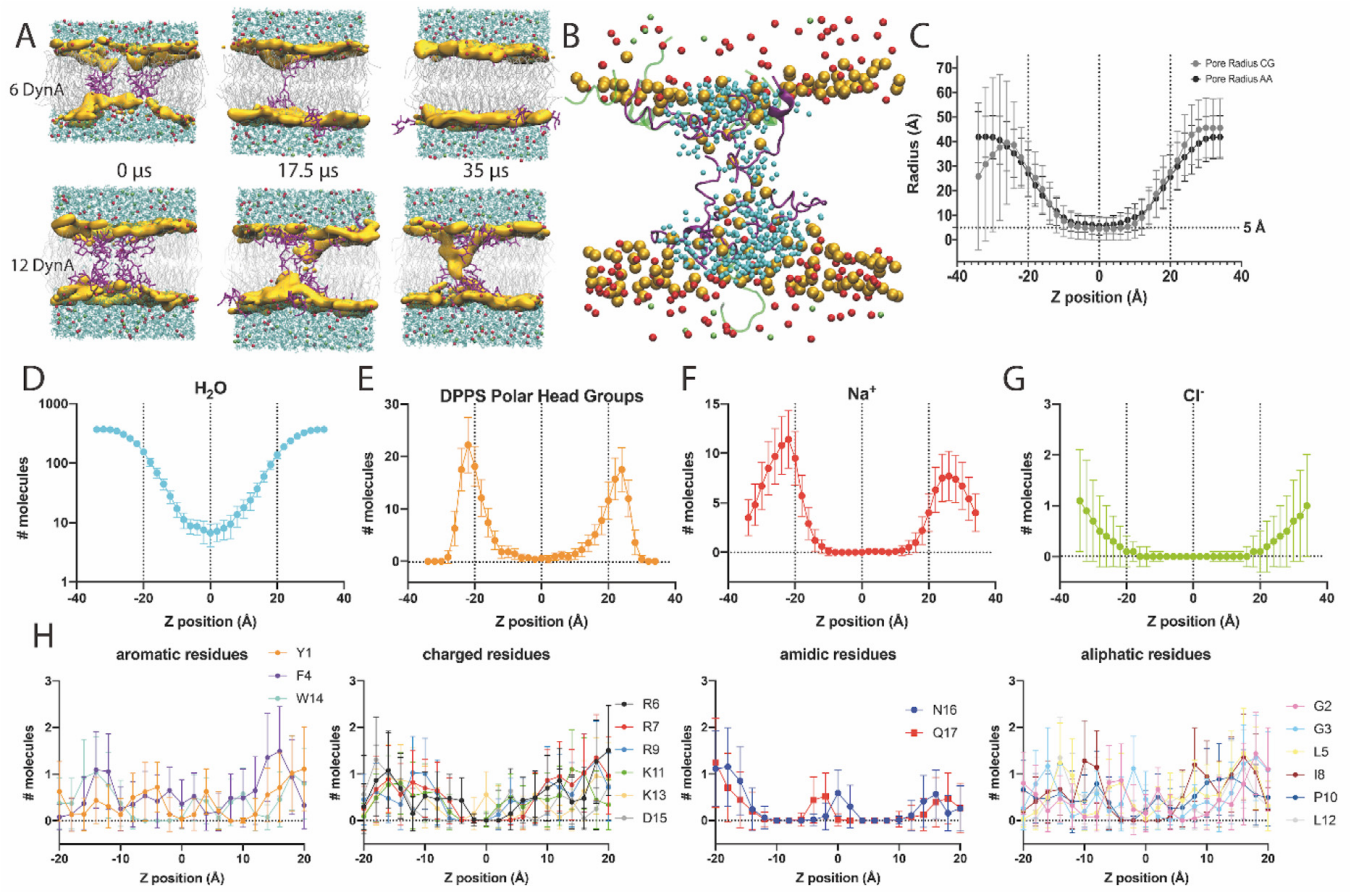
Molecular model of DynA pore formation. (A) Selected time points of the production step of CGMD simulations for the 6 DynA and 12 Dyn complexes. Water molecules are represented in cyan, peptides in purple, DPPS polar head groups in golden, DPPS tails in grey, sodium in red, and chloride in light green. (B) AAMD simulation of the 12 DynA complex at 2 µs. DynA pore-forming peptides are depicted in purple, and pore-excluded DynA peptides are depicted in transparent green. (C) Average pore-radius measurements from CGMD (35 µs) and AAMD (2 µs) simulations. Distribution of molecules in the AAMD simulation box across the Z-axis: waters (D); DPPS polar headgroups (E); sodium ions (F); chloride ions (G). Distribution of DynA residues in the AAMD simulation box across the Z-axis (H). (For interpretation of the references to color in this figure legend, the reader is referred to the web version of this article.)

**Fig. 6:**
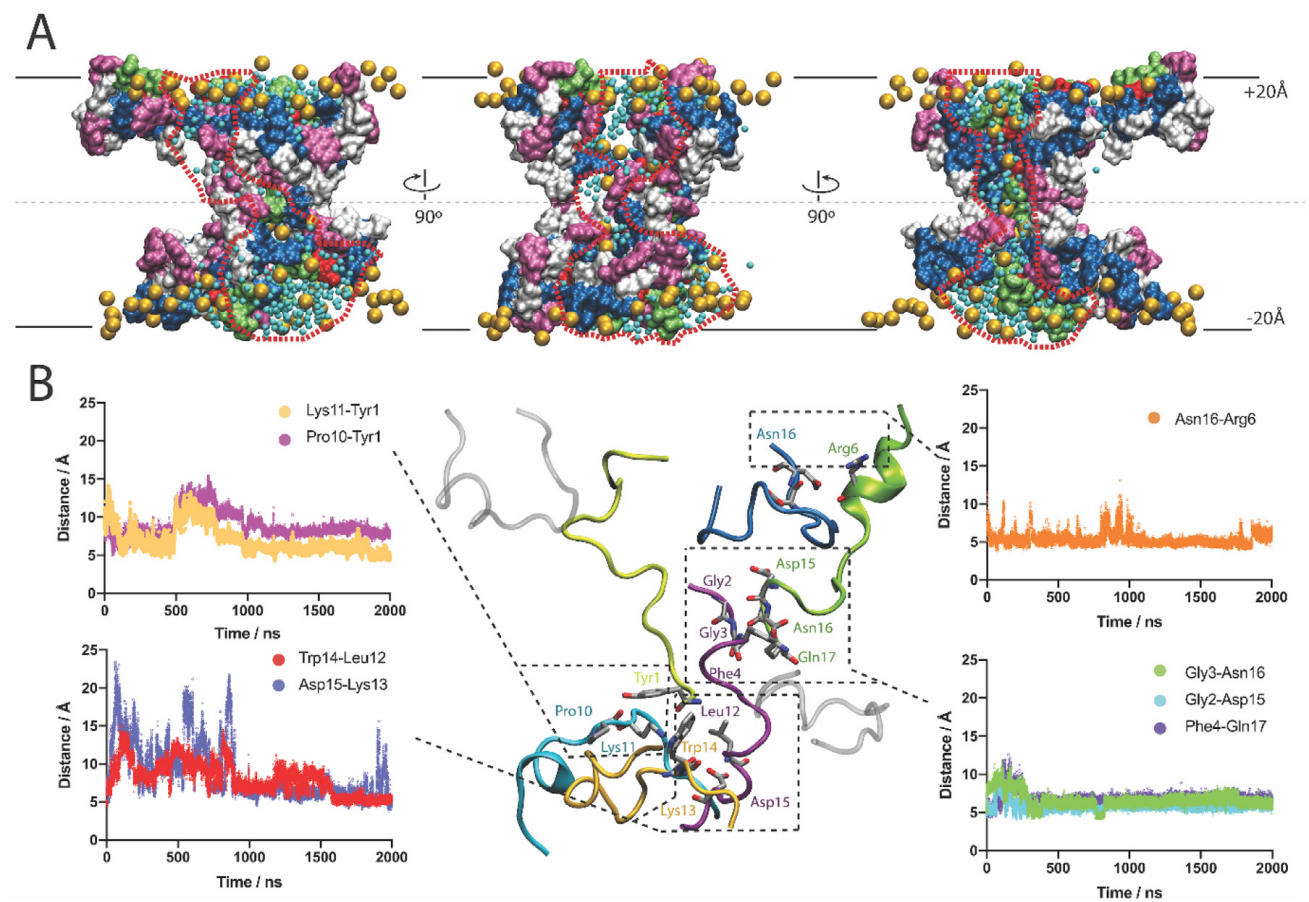
DynA-DPPS form non-structured proteo-lipidic pores. (A) Side views for the proteo-lipidic pore at 2 µs from the AAMD simulation highlighting the water pathway surrounded by a red dashed line. Phe, Tyr, and Trp are depicted in magenta; Gln and Asn are depicted in lime green; Asp is depicted in red; Arg and Lys are depicted in blue; Gly, Ile, Leu, and Pro are depicted in white. Water molecules are represented as cyan beads and DPPS polar head groups are depicted as golden beads. (B) Distance between residue pairs of DynA peptides within the DPPS bilayer, along the 2 µs trajectory. Residues in the same peptide unit are depicted in the same color as the peptide backbone. (For interpretation of the references to color in this figure legend, the reader is referred to the web version of this article.)

To gain molecular insights on how the amphipathic and positively charged DynA peptides interact with negatively charged membranes to form pores described in previous sections, we modeled the tridimensional structure of two DynA channel-like setups (6 DynA, and 12 DynA peptides) embedded in a DPPS bilayer (Fig. 5). Note that no particular emphasis has been done in the study of the protein/lipid ratio since conductance histograms show that there is not a predominant pore configuration but a myriad of them (Fig. 1). The 6 DynA and 12 DynA models were used as starting points for 35 µs CGMD simulations, which pursue to assess the partitioning of the peptide system in the lipid bilayer and the stability of the channel/peptides in the hydrophobic core of the bilayer. CGMD was performed without any applied voltage in line with electrophysiological recordings showing current fluctuations of equilibrium nature, consistent with pores formed spontaneously (Fig. 5). In the 6 DynA system all six DynA peptides transiently shifted from the bilayer to the aqueous phase in approximately 30 µs in the CGMD step (no peptide was in the bilayer at the end of the 35 µs CGMD, Fig. 5A). On the contrary, the 12 DynA system allowed the formation of a pore-like proteolipidic structure where DynA interacts with and bends DPPS polar heads inwards, bridging both sides of the bilayer (Fig. 5A). This could be one of the multiple conformations obtained using electrophysiology and exemplifies DynA ability to form proteolipidic pores. Following the CGMD, we ran a 2 µs AAMD simulation with the 12 DynA system toward obtaining qualitative molecular information regarding the pore, based on peptide-peptide and peptide-lipid interactions. The system remained stable during the 2 µs simulation, despite the increased temperature used compared to the CGMD simulation (323.15 K vs. 303.15 K). As shown in the AAMD simulation (Fig. 5B), 8 out of 12 peptides are cross-interacting with lipids forming a non-structured proteolipidic pore [30]. Peptides bridging the interfaces along the hydrophobic core are in extended conformation (Fig. 5B). The 12 DynA pore-like proteolipidic structure, both in CGMD and AAMD systems, has an average radius of ∼5 Å (Fig. 5C). Water molecules are preferentially partitioned in the water-bilayer interface (Fig. 5D), although discrete molecules penetrate the pore (Fig. 5B). On average, 104 water molecules are found inside the pore (within ± 10 Å) during the 2 µs AAMD simulation (Fig. 5D), which is comparable to the numbers reported for other peptide-formed pores like Alamethicin [74], [75] and much higher than in a closed state of the SARS-CoV-2 E proteolipidic pore [76]. Water molecules are located in all the positions along the Z-axis (Fig. 5D), contrary to what is found in closed ion channels due to hydrophobic gating [77], [78]. The peptide-free DPPS headgroups are distributed in the water-bilayer interface, but the peptide-contacting DPPS headgroups are forced towards the bilayer core (Fig. 5E and B). Na^+^ ions (Fig. 5F) follow the same distribution as the DPPS headgroups, but again, discrete Na^+^ ions can be found in the pore at specific time points in the trajectory (Fig. 5B), as opposed to Cl^-^ ions that are always distributed in the water region (Fig. 5G). Peptides partitioned at the water-bilayer interface are in a carpet-like fashion (Fig. 5B), with aromatic and charged residues (Fig. 5H) contacting with lipid headgroups (Fig. 5E) around the ± 20 Å region (bilayer thickness). Tyrosine 1 is crucial for DynA pore making potential [30] but during the MD simulation does not show a preferential position within the lipid bilayer. Closer to the hydrophobic core, arginine 6, 7, 9 and lysine 13 and 11, are distributed within ± 20 Å and ± 5 Å, respectively, probably due to the electrostatic interaction with negative charges of DPPS. Lys13 shows deeper distribution in the bilayer, below ± 5 Å, allowing Asn16, and Gln17, thus the DynA C-terminus, to sit in the hydrophobic core of the bilayer (Fig. 5H and Fig. 6), which are not the expected hydrophobic propensities for positively charged and amidic residues (Fig. 5H and Fig. 6). As reported in *Lind et al.* [17], the N-terminus of DynA can insert in bilayer-mimicking systems. In line with these data, Fig. 6B shows that two peptides have the N-terminus inserted in the bilayer (peptides depicted in light yellow and purple). The N-terminus of one of these peptides (Gly2, Gly3, Phe4 in the peptide depicted in purple, Fig. 6B) is interacting in an antiparallel fashion with the C-terminus of another DynA peptide (Asp15, Asn16, Gln17 in peptide depicted in green, Fig. 6B). To assess whether some interpeptide interactions are in place to maintain the pore, we have analyzed the distance along the 2 µs trajectory between specific residue pairs (Fig. 6B). Residue pairs in close contact (ca. 5 Å distance) are shown in Fig. 6B as representative of system packing allowing the stabilization of the pore complex. We have detected a stable putative salt bridge in close distance between Lys13 and Asp15 (peptides depicted in golden and purple, respectively, Fig. 6B), which does not prevent a salt bridge transitory mechanism to be important for this system, as it has been shown to be relevant for certain amyloid proteins [79].

At this point it is important to highlight that the residue distribution shown in our study using unconstrained peptides differs significantly from previous computational studies on single peptides in a secondary structure constrained conformation for native and pathogenic DynA variants in neutral lipid bilayers [80]. Single peptides in neutral membranes, such as POPC, rapidly follow White and Wimley water-interface/interface-bilayer core propensities and residues are distributed accordingly [80], [81]. Quite in contrast, peptide interactions with the negatively charged PS polar head allow the proteo-lipidic structure to stabilize towards the hydrophobic core in a steady pore-forming fashion (Fig. 5B and Fig. 6). Therefore, calculations are in line with experimental recordings described in previous sections showing intense ion-channel activity of DynA in charged membranes.

## 4 Conclusions

Electrophysiological recordings in planar bilayers show that DynA induces formation of pores in negatively charged membranes showing a remarkable diversity of conducting levels and lifetimes. Indeed, conductance histograms point to the existence of multiple pore configurations with dimensions around the nanometer in diameter. We show also that channel currents are ohmic and display equilibrium conductance fluctuations, what disregards electroporation as the dominant mechanism of pore formation. The predominant selectivity to cations found in selectivity experiments indicates that DynA-induced pores have a proteolipidic character, because DynA peptides are positively charged so that negative lipid charges are necessarily involved in the ionic discrimination exerted by the pores.

By using a hybrid approach combining Coarse-grain with all-atom molecular dynamics simulations we assess the pore-forming potential of DynA multipeptide proposing two DynA multipeptide models, one with 6 peptides (6 DynA) and other with 12 peptides (12 DynA) in different positions of a DPPS bilayer. Both assemblies may be just discrete representations of the plethora of proteolipidic conformations that DynA peptides adopt in negatively charged lipid bilayers, as shown in electrophysiological experiments. In 6 DynA, peptides migrate to both sides of the bilayer, and the full complex disassembles, but 12 DynA achieves a proteolipidic structure of at least 8 peptides, which lasts throughout the CG + AA simulation. We clearly show that DynA is capable of assembling in charged membranes to form a water-filled pore stabilized by lipid molecules. Simulations show that waters form hydrogen bridges along the pore, confirming its hydrophilic character and hence its potential ability to conduct ions.

The combination of experimental and computational data presented in this study provides new insights into the proteo-lipidic assembly of dynorphins as membrane perturbing peptides, especially regarding previously observed DynA membrane interaction and perturbation effects, leading to cation influx in cellular and *in vitro* systems, and even peptide translocation [17], [18], [19], [23]. Current knowledge on dynorphins include bilayer-induced secondary structure conversions, translocation potential [23], or liposome membrane perturbation in mixed phosphatidylcholine and phosphatidylglycerol compositions [18], [19]. To gain knowledge on the DynA pathophysiological mechanism, further experimental/computational combinatorial studies will be required, using state-of-the-art molecular dynamics methods to allow larger time scales (µs-to-ms) to observe secondary structure conversion events [16] and the use of asymmetric and customized ion and lipid compositions to assess cation selectivity and potential peptide translocation [23] *in silico*. In addition, our results and proposed methodology combining experimental electrophysiology measurements together with computational biophysics can be of particular interest to assess the pore forming potential of a large variety of compounds, such as viral proteins, antibiotic and bacterial lipopeptides, antimicrobial and/or cell-penetrating peptides, venom peptide toxins, and amyloid peptides [57], [63], [82], [83], [84].

## CRediT authorship contribution statement

**D. Aurora Perini:** Investigation, Formal analysis, Validation, Visualization, Writing – review & editing. **Marcel Aguilella-Arzo:** Methodology, Software, Investigation, Validation, Visualization, Writing – review & editing. **Antonio Alcaraz:** Conceptualization, Methodology, Validation, Writing – original draft, Supervision, Project administration, Funding acquisition. **Alex Perálvarez-Marín:** Conceptualization, Methodology, Validation, Resources, Visualization, Writing – review & editing, Project administration, Funding acquisition. **María Queralt-Martín:** Conceptualization, Methodology, Validation, Resources, Writing – original draft, Supervision, Project administration, Funding acquisition.

## Declaration of Competing Interest

The authors declare that they have no known competing financial interests or personal relationships that could have appeared to influence the work reported in this paper.
