# Supplementary material for: Dynorphin A induces membrane permeabilization by formation of proteolipidic pores. Insights from electrophysiology and computational simulations
Source: Comput Struct Biotechnol J. 2021 Dec 16;20:230–40. doi: 10.1016/j.csbj.2021.12.021 (PMC8718563; doi:10.1016/j.csbj.2021.12.021)
Supplement: Supplementary data 1 [file mmc1.docx]

**SUPPLEMENTARY FIGURES**

**
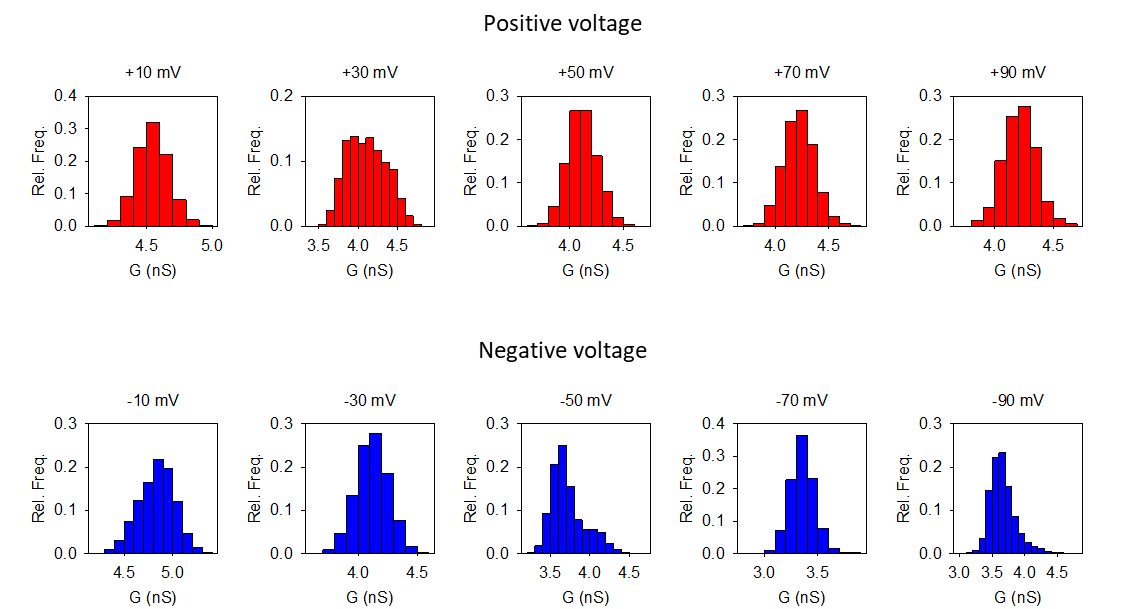
**

**Supplementary Figure S1. Current levels shown in Figure 3A can be represented by a single peak in a histogram.** Conductance histograms corresponding to each trace shown in Figure 3A for voltages ranging from ±10 to ±90 mV. For all explored voltages, all histograms display a single peak.
